# Understanding the significance of oxygen tension on the biology of *Plasmodium falciparum* blood stages: From the human body to the laboratory

**DOI:** 10.1371/journal.ppat.1012514

**Published:** 2024-09-19

**Authors:** Dinah S. Nahid, Kevin A. Coffey, Amy K. Bei, Regina Joice Cordy

**Affiliations:** 1 Department of Biology, Wake Forest University, Winston-Salem, North Carolina, United States of America; 2 Department of Epidemiology of Microbial Diseases, Yale School of Public Health, New Haven, Connecticut, United States of America; Joan and Sanford I Weill Medical College of Cornell University, UNITED STATES OF AMERICA

## Abstract

*Plasmodium falciparum* undergoes sequestration within deep tissues of the human body, spanning multiple organ systems with differing oxygen (O_2_) concentrations. The parasite is exposed to an even greater range of O_2_ concentrations as it transitions from the human to the mosquito host, suggesting a high level of plasticity as it navigates these different environments. In this review, we explore factors that may contribute to the parasite’s response to different environmental O_2_ concentrations, recognizing that there are likely multiple pieces to this puzzle. We first review O_2_-sensing mechanisms, which exist in other apicomplexans such as *Toxoplasma gondii* and consider whether similar systems could exist in *Plasmodium*. Next, we review morphological and functional changes in *P*. *falciparum*’s mitochondrion during the asexual-to-sexual stage transition and discuss how these changes overlap with the parasite’s access to O_2_. We then delve into reactive oxygen species (ROS) as ROS production is influenced by O_2_ availability and oxidative stress impacts *Plasmodium* intraerythrocytic development. Lastly, given that the primary role of the red blood cell (RBC) is to deliver O_2_ throughout the body, we discuss how changes in the oxygenation status of hemoglobin, the RBC’s O_2_-carrying protein and key nutrient for *Plasmodium*, could also potentially impact the parasite’s growth during intraerythrocytic development. This review also highlights studies that have investigated *P*. *falciparum* biology under varying O_2_ concentrations and covers technical aspects related to *P*. *falciparum* cultivation in the lab, focusing on sources of technical variation that could alter the amount of dissolved O_2_ encountered by cells during in vitro experiments. Lastly, we discuss how culture systems can better replicate in vivo heterogeneity with respect to O_2_ gradients, propose ideas for further research in this area, and consider translational implications related to O_2_ and malaria.

## Introduction

Malaria caused by *Plasmodium falciparum* can induce systemic hypoxia by disrupting blood flow and inducing loss of oxygen (O_2_)-carrying erythrocytes, leading to impairment of O_2_ delivery into deep tissue. The host’s response to hypoxia includes increased tissue expression of hypoxia-inducible factor (HIF) pathways, which are up-regulated during the pathogenesis in malaria and other infectious diseases [1–4] as well as in a range of noninfectious diseases including some cancers [5]. Although it is well understood how *P*. *falciparum* and other infectious agents affect the host at the level of reducing tissue oxygenation and inducing downstream HIF pathways, it is not well understood the extent to which varying O_2_ microenvironments may affect the parasite. This includes impacts on its population genetics, gene expression, epigenetic profile, posttranslational modifications, metabolic activity, and parasite multiplication rate (PMR), a metric used in *Plasmodium* studies to assess the per cycle fold-change in parasitemia to understand population-level parasite growth [6].

**Fig 1:**
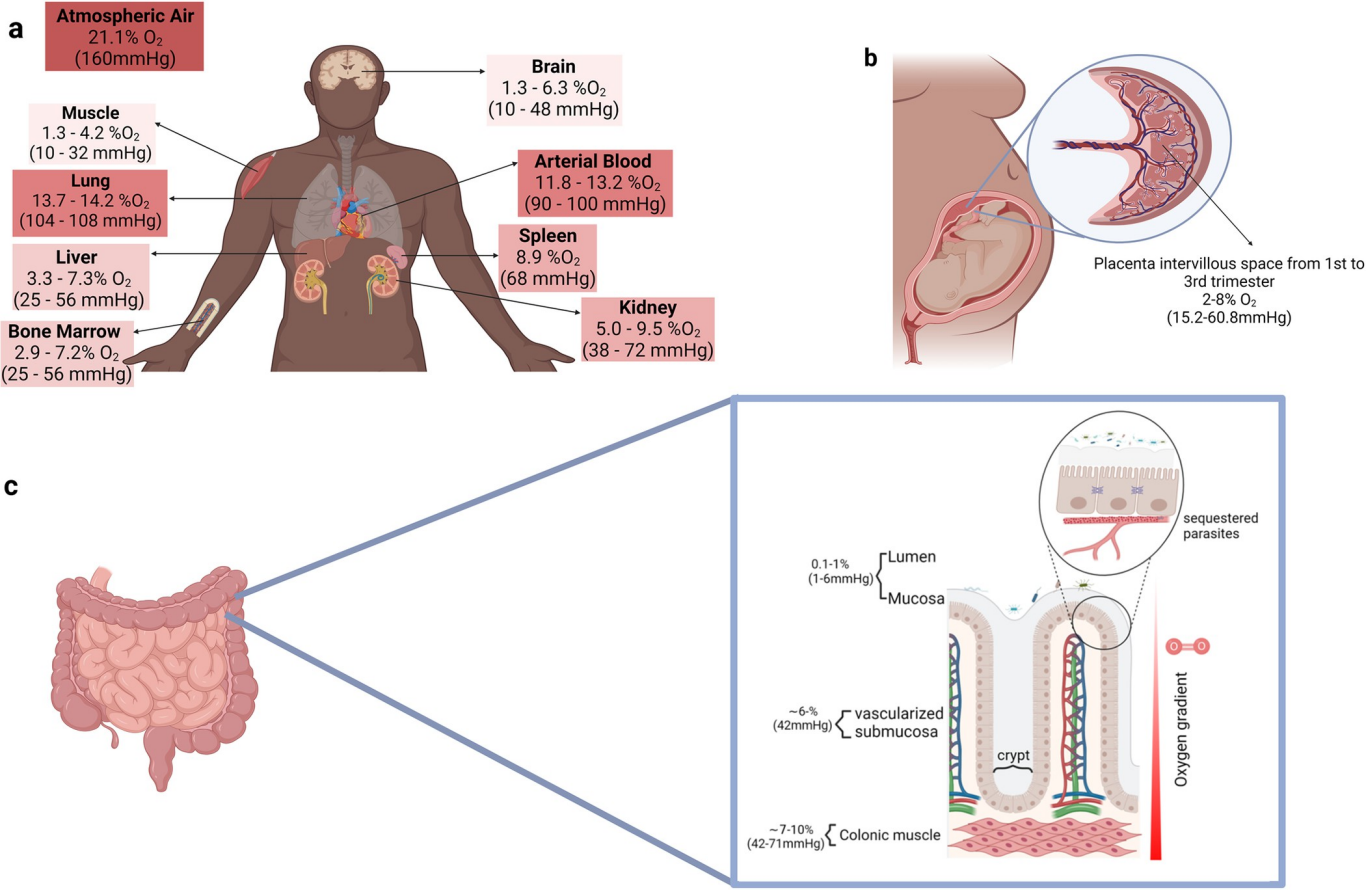
Variation in O_2_ concentration exists across and within organs of the human body. **(a)** Organ-level O_2_ values under physiological conditions, adapted from mean and median values reported in 4 comprehensive review articles on this topic: Jagganathan et al. [7], Ortiz-Prado et al. [8], Swartz et al. [9], and Gan and Ooi [85]. Shading is indicative of level of O_2_ concentration, with darker shading indicating higher O_2_ and lighter shading indicating lower O_2_. **(b)** Placental intervillous space O_2_ concentration in the location where parasite sequestration takes place during placental malaria. **(c)** O_2_ gradient in the layers of the gut, as discussed in Schwerdtfeger et al. [19]. In all panels, O_2_ concentrations are shown as both percentages and mm Hg and were calculated using the atmospheric partial pressure of O_2_ equation following Dalton’s Law in Ortiz-Prado et al. [8], which is as follows: _Atm_PO_2_ = FiO_2_ (fraction of inspired O_2_) × 760 mm Hg or _Atm_PO_2_ / 760 mm Hg × 100 = %O_2_.

*P*. *falciparum* parasites are exposed to a wide range of O_2_ concentrations during the course of their complex life cycle, which is spent between humans and mosquitoes, transitioning them between the highly oxygenated environment of the mosquito vector closely matching that of ambient air (approximately 20% to 21% O_2_) to the lower oxygenated environment of the human body with concentrations varying across different sites of the body from about approximately 1% to 14% O_2_ **(Fig 1)** [7,8]. In the literature, O_2_ tensions are reported as measures of partial pressure of O_2_ (in mm Hg) and as concentrations of O_2_, among other metrics. These are constantly in flux in the body, leading to considerable ranges in these values and are thus typically reported as mean or median values measured in an organ under physiological conditions [9]. The partial pressure of O_2_ is the amount of pressure exerted by O_2_ in a gas mixture, while the concentration of O_2_ is the amount of O_2_ in a gas mixture relative to other gases. The latter is a commonly used metric by researchers doing in vitro studies with the parasite, while the former is routinely used in clinical settings.

During malaria infections caused by *P*. *falciparum*, the parasitized RBC binds to the endothelium during the second half of its blood-stage development, sequestering within capillary networks of tissue across a range of organs and exposing the parasite to diverse microenvironments with respect to O_2_ tension. *P*. *falciparum* necropsy and autopsy studies have demonstrated a hierarchy of organ locations in terms of enrichment of sequestered parasite density going from highest to lowest as: brain, heart, lung, liver, kidney, and skin, in that order (note that spleen, gut, nor bone marrow were not included in these early histological studies) [10]. More recent studies utilizing immunohistochemistry and quantitative PCR-based methods applied to a larger number of *P*. *falciparum* autopsy cases revealed a similar organ distribution with a hierarchy going from highest to lowest parasitized as: spleen, brain, heart, gut, bone marrow, lung, skin, liver, and kidney [11], and ELISA-based studies applied to tissue homogenates found brain, gut, and skin as the most common and heavily parasitized tissues [12,13]. Collectively, these studies point to the brain as an organ that has been found to be highly parasitized in multiple studies of humans who died from malaria. While it must be noted that many of these studies specifically focused on cerebral malaria, it can also be observed that the brain has one of the lowest O_2_ tension microenvironments in the human body **(Fig 1)**. It is unknown, but an interesting possibility to consider, whether the O_2_ environment in the brain aids in promoting *P*. *falciparum* growth rate in that organ for this microaerophilic parasite [14].

It is well established that *P*. *falciparum* grows best under conditions of low O_2_. William Trager and James B. Jensen in their seminal work in 1976 establishing continuous *P*. *falciparum* culture, utilized carbon dioxide and nitrogen (CO_2_ and N_2_) gas mixtures containing either 5% O_2_ or 1% O_2_, and the candle jar method in which a flame was included inside a chamber to lower the percentage of O_2_ from ambient levels of 21% down to around 8% to 10% O_2_ [15]. Trager and Jensen found that the use of the 1% O_2_ exhibited the highest level of parasite multiplication and were the first to establish that *P*. *falciparum* is a microaerophile that grows best in conditions of low O_2_, a seminal observation that unlocked the continuous in vitro culture of *P*. *falciparum* [14].

Across the organs of the human body, parasites are exposed to microenvironments with O_2_ concentrations ranging from roughly 1% to 14% in a healthy individual, and lower in individuals experiencing illness-induced hypoxia. While bound to endothelial cells and sequestered within the capillaries of the brain, parasitized erythrocytes can experience quite low O_2_ tension, between 1.3% and 6.3% O_2_ (10 to 48 mm Hg), whereas when bound in arterial capillaries of the lung, they experience much higher O_2_ tension, around 13.7% to 14.2% O_2_ (104 to 108 mm Hg), as shown in **Fig 1A**. How *P*. *falciparum* parasites respond to growing in these different microenvironments during their trophozoite and schizont stages is not well studied, as most *Plasmodium* in vitro work is conducted at 0.5% to 5% O_2_.

The placenta is also a specialized organ that creates a unique niche that can introduce O_2_ variation to the parasite and is also the location of another severe form of the disease: placental malaria. The intervillous space of the placenta, which is where the parasite sequesters, undergoes changes in O_2_ concentration during pregnancy. The development of the placental microvasculature further along in pregnancy leads to increased blood flow toward this region and oxygenation needed for fetal development with a progression from 2% to 3% O_2_ (15.2 to 22.8 mm Hg) in the first trimester to 5% to 8% O_2_ (38 to 60.8 mm Hg) by the third trimester [16,17]. In the case of placental malaria, the parasite undergoes sequestration in the placenta microvasculature, leading to reduced O_2_ delivery to developing fetuses, one of the damaging effects of this form of the disease **(Fig 1B)**.

Another organ that has an abundant number of parasites according to histological studies is the gut [9–11]. The layers of the gut contain considerable O_2_ variation, going from a low O_2_ environment in the lumen and mucosa (0.1% to 1% O_2_), toward a midrange O_2_ environment of the vascularized submucosa (6% O_2_), and finally reaching its highest levels at the colonic muscle (7% to 10% O_2_). Since *P*. *falciparum* parasites are found to be sequestered within capillaries across different depths of the gut, although primarily in the vascularized submucosa and colonic mucosa, they are exposed to some range of O_2_ concentration in that organ **(Fig 1C)**. The gut O_2_ gradient is well known to influence the distribution of gut microbiota, with primarily anaerobic bacteria that prefer low to no O_2_ residing in the lumen and mucosa [18,19].

The ability of *P*. *falciparum* to withstand such a high degree of variation in O_2_ tension demonstrates a high level of plasticity of this organism not only to survive and replicate within a single host but also for it to be successfully transmitted between the human and mosquito host. In the following sections, we review 4 areas that may contribute to the plasticity of this parasite as it survives across a range of O_2_ tensions: (i) an O_2_-sensing mechanism as is seen in other related protozoa; (ii) morphological and functional changes in the *P*. *falciparum* mitochondrion during its life cycle transitions; (iii) reactive oxygen species (ROS) and how oxidative stress impacts the parasite; and (iv) oxygenation state of hemoglobin within the host RBC and how this impacts the parasite. We also review technical components of parasite cultivation in relation to O_2_, the various culture containment devices used in parasite maintenance, and implications of these methods on our understanding of the parasite’s biological processes.

### Oxygen sensing in parasitic protozoa and implications for *P*. *falciparum*

When trying to understand the effects of O_2_ variation on *P*. *falciparum*, it is worth considering how O_2_ affects other protozoan parasites and what mechanisms mediate their ability to sense and respond to differences in O_2_. In many eukaryotic organisms, O_2_ sensing is facilitated by the HIF pathway. In these organisms, cells regulate the expression of hypoxia genes by modifying the HIFα transcription factor under normoxic and hypoxic conditions. In the presence of O_2_, the O_2_-dependent degradation domains (ODD) on HIFα are hydroxylated by the prolyl hydroxylase domain (PHD) enzymes and factor inhibiting HIF (FIH) and then tagged with von Hippel–Lindau tumor suppressor and ubiquitinated for proteasomal degradation. In hypoxic conditions, HIF is not hydroxylated and remains stable to enter the nucleus, bind with HIFβ, and begin transcription of hypoxia genes [20,21]. Genomic analysis of protists reveal a lack of canonical HIF pathways in these organisms, including for apicomplexan parasites like *Plasmodium* spp. and *Toxoplasma* spp. [22–24]. The expression of PHDs can occur independently of HIFs with many systems lacking HIF pathways while still containing PHD enzymes. These regulatory elements are shown to have evolved prior to HIFα [23] and have been shown to be directly responsible for cellular O_2_ sensing in cells and organisms lacking HIFs.

In their thorough review on this subject, West and Blader discuss the intricacies of the O_2_ sensing on protozoan pathogens, from amoebas to apicomplexans [22]. *Dictyostelium discoideum* is a soil-dwelling amoebae that has the capacity to undergo aerotaxis, which is the movement toward environments with higher O_2_ concentrations from lower oxygenated environments [25]. The PHD enzyme that *D*. *discoideum* possesses, *DdPHYa*, is also conserved in *Toxoplasma gondii*, where it is called *TgPHYa*. Additionally, the DdSKP1-modifying glycosyltransferases are also conserved in *T*. *gondii* [26–28]. *T*. *gondii* parasites survive in low O_2_ concentrations when they first infect hosts via the gastrointestinal tract and, like malaria parasites, are then exposed to a wide range in physiological O_2_ over their life cycle, as the tachyzoite stages relocate to organs across the human body, ranging from around 1% to 6% O_2_ in the brain to around 14% O_2_ in the lungs [3,22]. *TgPHYa* is important for the growth of *T*. *gondii* in lower O_2_ concentrations [26]. In addition, a separate class of PHDs called *TgPHYb’s* also serve as O_2_ sensors for higher O_2_ concentrations and regulate translation elongation during protein synthesis [29]. Interestingly, *Plasmodium* lacks a *PHYa* orthologue but has a putative *PHYb* PHD subclass [29]. However, it is unclear if the *Plasmodium PHYb* functions in the same capacity as that of *T*. *gondii*. More work needs to be done to determine whether O_2_ sensing through a similar pathway as *T*. *gondii* exists at all in *Plasmodium*.

### Structure and function of the mitochondrion across the *P*. *falciparum* life cycle

**Fig 2:**
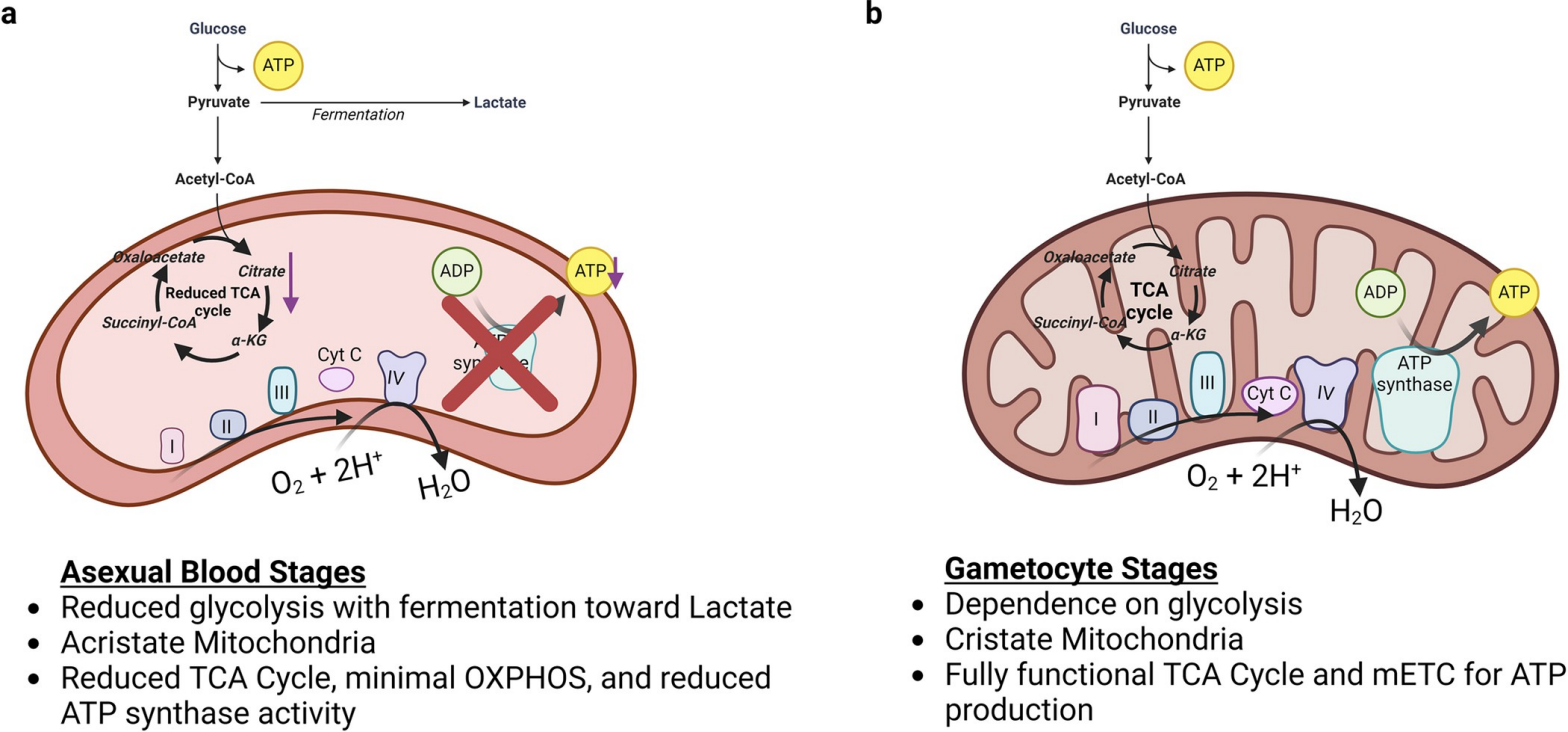
Variation in the structure and biochemical pathway activity of *Plasmodium*’s mitochondrion exists between asexual and sexual blood stages. Mitochondrial internal structure, glycolysis, mETC, and OXPHOS capacity variation between the (a) asexual blood stage and (b) the gametocyte stage. mETC, mitochondrial electron transport chain; OXPHOS, oxidative phosphorylation; TCA, tricarboxylic acid.

As in other eukaryotes, O_2_ consumption takes place in the mitochondrion of *Plasmodium*, making it an organelle of great interest in our understanding of how the parasite may be affected by variation in O_2_ concentration. The solo mitochondrion of *P*. *falciparum* undergoes a great deal of morphological change during the life cycle of the parasite, which may also be associated with differences in function [30–32]. During the asexual blood stage, the mitochondrion is acristate, whereas cristate structures are formed during gametocytogenesis, leading to the presence of cristae during gametocyte development, ultimately ending up in the O_2_-rich environment of the mosquito **(Fig 2)** [33]. Cristae are a physical indicator of a mitochondrion being capable of aerobic metabolism, whereas acristate mitochondria are typically characteristic of obligate anaerobes [34]. Mitochondrial cristae contain the majority of the mitochondrial electron transport chain (mETC) complexes and ATP synthase, which suggests that the acristate form may have a markedly reduced tricarboxylic acid (TCA) cycle progression and oxidative phosphorylation (OXPHOS) capacity [35]. The gametocyte stages have also been shown to have a higher abundance of TCA cycle and OXPHOS associated proteins than blood-stage parasites [35]. The proposed means of ATP production in both asexual blood stage and gametocyte stage development is also shown in **Fig 2**.

The TCA cycle metabolism has been shown to be not required for erythrocytic development of *Plasmodium* parasites, which primarily depend on glycolysis for generating ATP. Work by Ke and colleagues [36] using *P*. *falciparum* in human RBCs compellingly demonstrated that mitochondrial ATP synthesis is not required for the parasite to replicate in erythrocytes but that it is required for the parasite to transmit successfully to mosquitoes. This work was done using knockout (KO) strains for 6 of 8 TCA cycle enzymes of *P*. *falciparum* and showed no impairment in multiplication compared to the wild-type strain in asexual erythrocytic development but did show a block in the development of gametocytes into oocysts in the mosquitoes in one of the KO strains [36]. A study done by Rajaram and colleagues showed that the remaining 2 TCA cycle enzymes, malate-quinone oxidoreductase (MQO) and fumarate hydratase (FH), are also nonessential in the asexual stages as shown by the fitness of the MQO and FH deletion strains they generated and tested [37]. Additionally, studies by Sturm and colleagues using a *Plasmodium berghei* model of malaria in mice employed a mitochondrial ATP synthase β subunit gene KO strain and showed only a marginal difference in asexual parasite replication but observed defects in in vivo ookinete development and the prevention of oocyst and sporozoite formation [38]. These defects include a greater presence of aberrant females, reduced activated females, reduced zygote stages, and a significant reduction in ookinetes [38]. These results suggest that the parasite is sensitive to ATP synthase loss and that there may be a greater role of the TCA cycle during the mosquito stages of development.

These studies demonstrate the lack of a requirement for the TCA cycle in erythrocytic development within the mammalian host but a necessity of the TCA cycle within the mosquito host, while also emphasizing the level of plasticity needed by the parasite in its energy metabolism for its successful propagation. Whether *P*. *falciparum*’s plasticity also plays a role as the parasite moves between diverse niches of the human body in terms of O_2_ tension is unknown, but of interest to explore given the range of O_2_, glucose, and other plasma metabolites that vary substantially in vivo as compared to controlled in vitro settings [7,39,40]. Few studies have directly measured OXPHOS activity in *P*. *falciparum* in response to such environmental changes. However, a study by Sakata-Kato and colleagues investigated O_2_ consumption and mitochondrial respiration in *P*. *falciparum* in response to changes in environmental O_2_ and glucose using an extracellular flux analyzer. The authors demonstrated that a depletion in glucose, the first metabolite in the glycolysis pathway, can facilitate a switch between glycolysis and mitochondrial respiration with low glucose levels leading to a shortage of energy necessitating an increase in O_2_ consumption and OXPHOS [41]. Given these findings, it is interesting to consider the possibility that the parasite may compensate for energy deficits that may occur in vivo over the course of infection; however, this area of research needs further investigation to determine whether those links exist.

### Sources and roles of reactive oxygen species (ROS) in *P*. *falciparum* infection

**Fig 3:**
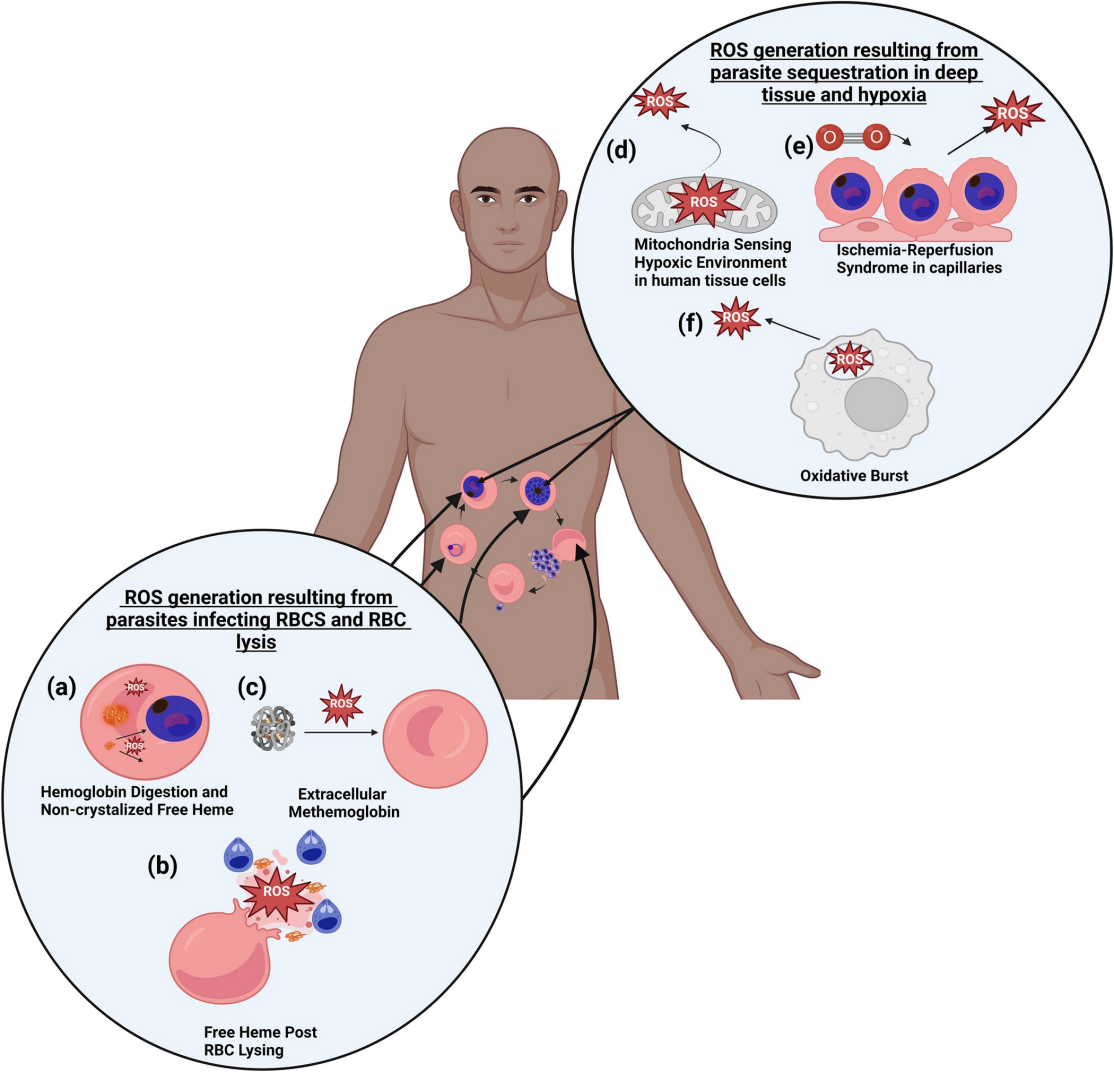
Sources of reactive oxygen species (ROS) during malarial infection. ROS generation resulting from parasites infecting RBCs and from RBC lysis (left panel, **a-c**) and ROS generation resulting from parasite sequestration in deep tissue and from hypoxia (top right panel, **d and e**). **(a)** *P*. *falciparum* metabolizes hemoglobin inside the RBC and converts heme to hemozoin for detoxification. Excess heme iron that escapes the conversion into hemozoin can promote production of the hydroxyl radical (·OH) inside the parasitized RBC. **(b)** Free heme is released into the blood plasma during the parasite-mediated lysis of RBC, where it induces ROS in a variety of cell types across the body. **(c)** Extracellular methemoglobin which is in the plasma of individuals with severe malaria can induce oxidative stress within uninfected RBCs causing them to externalize phosphatidylserine and aggregate. **(d)** Mitochondria experience changes in tissue oxygenation, resulting in ROS production. **(e)** Ischemia-reperfusion syndrome, which occurs as a response to reoxygenation following tissue ischemia, may lead to localized production of ROS. **(f)** Immune cells that have phagocytized infected RBC producing ROS as part of the oxidative burst response.

In glycolytic asexually replicating blood-stage *P*. *falciparum* parasites, the destruction of hemoglobin is both a key source of nutrients for the parasite but also a large source of ROS. *P*. *falciparum* digests hemoglobin from the host’s RBC, producing free heme-iron that damages both the parasite and RBC. The parasite mediates this oxidative assault by crystalizing heme molecules into hemozoin [42]. However, excess heme can fail to crystallize, escape hemozoin conversion, and subsequently cause oxidative stress within the cell, as depicted in **Fig 3A**. There is also a buildup of free heme that gets released after the parasite lyses the RBC post-schizogony, causing oxidative damage to uninfected RBCs and other nearby cells and tissue [42–46] **(Fig 3B)**. Another consequence of hemolysis is the release of free hemoglobin that can also auto-oxidize into methemoglobin, the oxidized form of hemoglobin in which heme-iron is in the ferrous (Fe^2+^) rather than the ferric state (Fe^3+^) alongside the release of superoxide (O^2⨪^). Circulating methemoglobin induces oxidative stress on uninfected RBCs, which causes cell aggregation and can, in turn, result in the production of more methemoglobin within those cells [47] **(Fig 3C)**.

Beyond the extracellular impacts of free heme and its downstream impacts, *P*. *falciparum* sequestration itself can drive the onset of not just local ischemia but also a response from the host that drives increase ROS production. As the number of infected RBCs sequestering within a particular capillary increases, blood flow can become blocked, leading to localized ischemia [48]. In prolonged exposure of hypoxia, the host mitochondria decrease the production of ATP and begin degrading it into adenosine diphosphate, adenosine monophosphate, and then adenosine. Adenosine can revert into ATP if O_2_ becomes available; otherwise, it will irreversibly metabolize to inosine and hypoxanthine. If reoxygenation occurs after hypoxanthine is synthesized, hypoxanthine is converted into xanthine along with the production ROS [49] **(Fig 3D)**. The ischemia can ultimately be reversed but has the potential for ischemia-reperfusion syndrome (IRS), which can lead to a striking increase in ROS as O_2_ reenters a formerly hypoxic tissue site upon recovery [43] **(Fig 3E)**.

The host immune system is another source of ROS within the human body during a malaria infection. Monocyte-derived macrophages (MDM) and dendritic cells are recruited by xanthine oxidase enzymes to stimulate a response by causing oxidative stress and inflammation in the host in the presence of *P*. *falciparum*-infected RBCs. The activation of MDMs causes these cells to secrete additional inflammatory cytokines such as IL-1ß [50–52]. Additionally, phagocytic cells including macrophages and neutrophils engulf infected RBCs and transfer them into the phagosome where they induce a process called oxidative burst, where they use NADPH oxidase to kill the phagocytosed parasite. Furthermore, some ROS molecules exit the cell and damage nearby cells during this process as well [53] **(Fig 3F)**.

### Hemoglobin oxygenation state and its impacts on *P*. *falciparum* digestion

It is well established that *P*. *falciparum* takes hemoglobin into its digestive vacuole, where aminopeptidases break it down and release amino acids so that the parasite can use them for its own growth. What is not as well studied is to what extent the oxygenation state of hemoglobin impacts the parasite’s ability to digest and process it. Given that *Plasmodium* parasites reside within O_2_-carrying erythrocytes and that they rely on digesting hemoglobin for their survival and growth, it may very well be that the oxygenation state of the hemoglobin itself could have a large impact on the parasite’s biology and growth. Hemoglobin’s primary function in the body is to transport O_2_ from the lungs to the deep tissues (the O_2_ in this case being used primarily to fuel mitochondrial OXPHOS in human cells across the body), and it also carries carbon dioxide (CO_2_) from the deep tissue back to the lungs. This process occurs through the binding and release of O_2_ and CO_2_ from hemoglobin, and as a result, hemoglobin is found within the human body in varying amounts of oxyhemoglobin (Hb bound to O_2_), carboxyhemoglobin (Hb bound to CO_2_), deoxyhemoglobin (unbound Hb), and methemoglobin (irreversibly unbound Hb) [54]. Methemoglobin is produced, as mentioned above, due to oxidation of the iron ion of hemoglobin, oxidizing it from the ferrous (Fe^2+^) to the ferric state (Fe^3+^), rendering it permanently unable to bind O_2_. Several studies have shown that there is elevated levels of methemoglobin in the blood of malaria patients, particularly in those with severe malaria [55]. What is interesting, however, is that 2 in vitro studies have demonstrated that *P*. *falciparum* parasites may preferentially digest methemoglobin over oxidized hemoglobin. These studies show that (i) one of *P*. *falciparum*’s enzymes for degrading hemoglobin, falcipain-2, binds methemoglobin at a significantly higher affinity than it does hemoglobin [56] and that (ii) a reduction in methemoglobin induced through riboflavin led to a reduction in parasite food vacuole size and in PMR [57]. Given these findings, it is plausible that the parasite may respond indirectly to changes in microenvironmental O_2_ levels through its interaction with different forms of hemoglobin—growing faster or slower depending on the ratios of oxidized versus methemoglobin in its host cell. More research is needed to fully explore these potential interactions.

### Connections between antimalarial mechanisms and oxidative stress

In addition to the impacts of O_2_ concentrations on the parasite and/or its host cell, studies evaluating the in vitro efficacy of antimalarial drugs have revealed differences in activity depending on the level of O_2_ in the microenvironment. Many antimalarial drugs have mechanisms of action that include the induction of oxidative stress in the parasite. Atovaquone, quinolines, and artemisinin and its derivates either directly or indirectly generate ROS that contribute to parasite killing [58]. Additionally, *P*. *falciparum* glutathione antioxidant activity is involved in some mechanisms of antimalarial drug resistance [59]. Beyond ROS activity on the parasites, variability in culture conditions including the O_2_ concentrations in the microenvironment can impact the overall efficacy of antimalarial drugs in vitro by mechanisms independent of target-site mutations. A landmark study demonstrated that parasites cultured in an oxidative stress microenvironment were less sensitive to dihydroartemisinin (DHA) treatment than control parasites that were not preexposed to oxidative stress [60]. Similar trends have been described for O_2_ concentration in culture impacting in vitro susceptibility to chloroquine [61], artemisinin, and lumefantrine [62]. Such findings underscore the impact of the role of O_2_ and ROS in the microenvironment and the potential impact on antimalarial efficacy. Such findings are intriguing and emphasize the need for further studies to explore how such differences in the in vitro oxygenation status translate to in vivo O_2_ microenvironments and the precise impact on antimalarial drug activity and therapeutic efficacy.

### Technical considerations in *P*. *falciparum* cultivation as it relates to oxygen

Great strides have been made in malaria research since Trager and Jensen’s classic publication regarding the long-term cultivation of *P*. *falciparum*. However, questions still exist about what role(s) O_2_ plays in the growth and development of *P*. *falciparum* blood-stage parasites. In this section, we discuss the technical aspects of O_2_ exposure related to *P*. *falciparum* cultivation in the lab and how this influences our studies on the biology of the parasite, including the measurement of ambient versus dissolved O_2_ in media, the measurement of oxygenated versus deoxygenated hemoglobin in RBCs, the variation in O_2_ concentrations administered to the chambers used for studies, the range of incubator types used, chambers, and cell culture vessels used, among other sources of technical variation that could alter the amount of dissolved O_2_ that is encountered by the cells in culture during in vitro experiments.

### Establishment of the *Plasmodium* culture system

When establishing continuous *P*. *falciparum* culture, Trager and Jensen utilized a gas mixture of 5% O_2_, 7% CO_2_, and 88% N_2_, a mixture of 1% O_2_, 7% CO_2_, and 92% N_2_, and the candle jar method [15]. Trager and Jensen found that use of the 1% O_2_, 7% CO_2_, and 92% N_2_ exhibited better parasite multiplication. They utilized the FVO strain of *P*. *falciparum* cultured with RPMI 1640, NaHCO_3_, Human AB+ serum, and Human AB+ blood and maintained the culture for over 2 months. Another group that independently achieved long-term in vitro parasite cultivation around the same time as Trager and Jensen did so by administering low O_2_ tension as well [63]. Further work done by Schiebel and colleagues established the microaerophilic nature of the parasite, which was a key finding for successful long-term in vitro culture of *P*. *falciparum* [14]. In this study, a series of gas mixtures were tested with alterations to the O_2_ and CO_2_ percentages with the main conclusions supporting culture utilizing lower O_2_ percentages. Even earlier culture work led to the removal of leukocytes from whole blood to prevent in vitro parasite mortality and enabled continued longer-term replication of parasites within erythrocytes in culture [64].

### Methods for culturing *Plasmodium falciparum*

The most utilized O_2_ concentrations for in vitro *P*. *falciparum* cell culture maintenance range from 1% to 5% O_2_ via custom gas mixes also containing 5% CO_2_ and balanced N_2_. There are also groups utilizing candle jar desiccator chambers where a lit candle goes out upon O_2_ depletion has been shown to be an affordable and effective way of maintaining parasite cultures [65]. Although affordable, the percentages of the gas composition cannot be directly controlled, and O_2_ percentages range from 14% to 18% O_2_. An alternate approach is to utilize a custom premixed cylinder with an O_2_ percentage of their choosing, 5% CO_2_ and balanced N_2_ into a modular incubator chamber that gets placed into an incubator. The use of a gas mixer connected to 100% O_2_, N_2_, and CO_2_ medical grade gas tanks allowing for manual adjustments to O_2_, N_2_, and CO_2_, has also been utilized with success for in vitro cultivation of clinical isolates and lab strains in field sites where access to a direct custom-gas mix is a major challenge [66]. Administration of the custom gas from a gas mixer has shown similar parasite growth rates in vitro than parasites given a custom-mix from a singular premixed cylinder.

Other options for containment devices include the use of flasks that can have a tubing line connected to a gas tank and serological pipet fed directly into the flask or containment in a tri-gas incubator capable of utilizing ambient O_2_ with 100% N_2_ and 100% CO_2_ tanks to obtain any desired decrease in O_2_ percentage. This system results in the rapid depletion of the connected N_2_ tank, however, necessitating having backup tanks or consistently scheduled deliveries of new N_2_ tanks for routine culture use in hypoxic conditions. N_2_ generators can also be utilized to accommodate the fast usage rate associated with maintaining a lower O_2_ with this system. A standalone 5% CO_2_ incubator has also been utilized with success for short-term parasite cultivation [67,68]. Ambient air supplies the O_2_ within this type of incubator and contains 20% to 21% O_2_. The incubator environment reduces this percentage to approximately 18.6% O_2_. This is due to the ambient air reaction to the incubator CO_2_ and H_2_O droplets from the water pan providing humidity within these incubators, inducing matching O_2_ levels within the same range as the candle jar method [69]. Whether there is an ideal O_2_ percentage for in vitro parasite growth has been a topic of debate in the field [70,71]; however, what is clear is that a range of O_2_ percentages are routinely utilized for parasite cultivation.

### Oxygen diffusion into liquid media

Another factor worth considering is the amount of O_2_ that dissolves in culture medium and reaches the parasites following the gassing process. It is well documented that O_2_ diffusion through culture media in other cell culture systems leads to a further decrease in O_2_ reaching cells [69,72]. To our knowledge, measuring dissolved O_2_ concentration in vitro is not routinely performed for *P*. *falciparum*. For other cell culture systems, an in vitro probe has been utilized to check dissolved O_2_ concentration, which could be helpful for malaria research and in understanding how extensively parasitized erythrocytes are being oxygenated following the transition from gaseous to liquid environments [73]. In general, little is known of how extensively these gas mixtures reach erythrocytes in cultures, especially given the use of different containment devices for routine culturing *Plasmodium*.

Many researchers utilize modular incubator chambers that can have gas directly administered into them via a tubing line connected to a gas cylinder. This method allows for parasite culturing in multiple petri dishes and multi-well plates with a uniformity in the gaseous atmosphere administered to those cultures. In general, these chambers are regarded as one of the best methods for maintaining the needed environmental O_2_ levels. In other cell culture systems, it is well established that shaking the cultures on an orbital shaker or shaking incubator improves O_2_ diffusion through the liquid media to the cells, but the extent and quantification of this diffusion for *P*. *falciparum* culture methods is not yet established. Our current understanding of in vitro cell culture O_2_ diffusion is mostly grounded in adherent cell line culture studies, whereas *P*. *falciparum* blood-stage cultures are nonadherent. For *P*. *falciparum* cultures, gentle shaking has primarily been viewed as a means of improving merozoite access to uninfected erythrocytes, thereby reducing the amount of multiply infected cells. Shaking also ensures a more even distribution of waste products from the parasites in the culture media with an inherent assumption of proper O_2_ diffusion [74,75], although this has yet to be explicitly studied to our knowledge.

It is possible to use an O_2_ meter within a modular incubator chamber to monitor the atmospheric O_2_ percentage within this contained system, but that only shows the atmospheric percentage and not the dissolved O_2_ percentage within the liquid culture medium. If the dissolved O_2_ concentration in cultures is lower than that of the air in their atmosphere, it could mean that previous studies on the PMR and changes in parasitemia under differing O_2_ concentrations were done in lower O_2_ values than originally thought. This could also mean that the utilization of more hyperoxic O_2_ percentages associated with the candle jar and standard atmospheric O_2_ may translate to lower O_2_ concentrations in culture, matching the physiological O_2_ range needed for parasite growth.

PMR and parasitemia have been utilized previously to gauge parasite response to O_2_ variation, along with cycle length. Torrentino-Madamet and colleagues found that parasites underwent prolonged schizogony when grown in 21% O_2_ compared to those grown in 5% O_2_ without a significant difference in parasitemia observed [76]. Others have also found no significant difference in parasitemia across differing O_2_ percentages [61,77]. Another study comparing parasite multiplication across different O_2_ concentrations utilized PMR to reflect the fold-change in parasite numbers, which was also shown to not differ [78]. **Table 1** shows select examples of studies in which different O_2_ concentrations have been compared, which percentages they compared, what containment devices were utilized in these studies, and how they quantified parasites as mentioned in their methods. While not an all-encompassing table, it is clear that variation has been seen in the growth rates of *P*. *falciparum* across different O_2_ conditions but that the results are not entirely consistent across all studies. More controlled experiments can help to better understand the actual role of dissolved O_2_ on *P*. *falciparum* growth and biology.

**Table 1:** Compilation of studies where different O_2_ concentrations were tested on parasite growth.

Study	O_2_ concentrations tested and culture containment used	Strains used	Study duration	Parasite quantification method	Parasite growth finding
Trager and Jensen 1976 [15]	1% and 5% O_2_ with parasites being cultured in a flow vial system (flat-bottomed vial connected to an overflow flask and reservoir flask containing fresh media) with O_2_ gas mixture administered to vial and reservoir flask. A culture was grown in 5% O_2_ for 36 days before being switched to 1% O_2_ and another culture was given 1% O_2_ for the entire duration of study. Also utilized candle jar.	FVO	Approximately 50 days (25 cycles)	Microscopic quantification of blood smears	Higher parasitemias observed in parasite culture being given 1% O_2_ for the whole study duration even though cultures were passaged throughout. Also observed excellent parasite multiplication in candle jar.
Scheibel et al. 1979 [14]	0.5%, 1%, 3%, 5%, 21% with petri dish cultures placed in a dessicator capable of having direct gas mixture administration. Candle jar also tested.	FCR3/FMG	120 hours (approximately 3 cycles)	Microscopic quantification of blood smears	Most optimal growth observed at 3% O_2_ compared to all other percentages tested and candle jar.
Briolant et al. 2007 [61]	5%, 10%, and 21% O_2_ were tested with no specification of culture device used and how gas concentrations were administered. A Forma series II Water-jacketed CO_2_ incubator was utilized.	3D7 and W2	Approximately 75–85 hours (approximately 2 cycles)	Microscopic quantification of blood smears.	No differences in parasitemia observed between 5%, 10%, and 21% O_2_.
Torrentino-Madamet et al. 2011 [76]	5% and 21% with no specification of culture device used and how gas concentrations were administered. They used a Forma series II Water-jacketed CO_2_ incubator utilized.	3D7	78 hours (approximately 2 cycles)	Microscopic quantification of blood smears	Prolonged parasite schizogony in 21% O_2_ compared to 5% O_2_, but no significant difference in parasitemia observed.
Archer et al. 2018 [78]	1%, 3%, 5%, 7.5%, 10%, and 16% O_2_ were tested for parasite growth in HbAA (erythrocytes from individuals with normal hemoglobin) and HbAS erythrocytes (from individuals who have the sickle cell mutation). Cultures were in petri dishes placed into a modular incubator chamber placed into an incubator (model unspecified).	D10	64 hours for PMR calculations (approximately 2 cycles), 44 hours for DNA content quantification (approximately 1 cycle)	Microscopic quantification of blood smears for PMR deductions and DNA content quantification by flow cytometry	No significant difference in PMR across all O_2_ percentages tested within HbAA. Equivalent amounts of DNA replication demonstrated via flow cytometry of DNA content in HbAA.
Crispim et al. 2022 [77]	Atmospheric air in a cell culture flask, atmospheric air in a glass bottle, and 5% O_2_ in a cell culture flask. Cultures placed into a MaxQ 6000 Shaking Incubator-4353 Model from Thermo Fisher. Gas concentrations were administered directly into glass bottle and flasks.	3D7, K1, and NF54	6 days (3 cycles)	Microscopic quantification of blood smears	Observed slightly more growth in atmospheric air than in 5% O_2_.

### Quantifying ratios of hemoglobin species in the culture

Taking this one step further beyond measuring dissolved O_2_ in the media, it is also possible to measure the O_2_-carrying state of the hemoglobin that is present within the RBCs of a parasite culture. With a colorimetric plate reader to capture measurements at specific wavelengths, it is possible to measure the amount of oxyhemoglobin, deoxyhemoglobin, carboxyhemoglobin, and methemoglobin in a bulk population of RBCs from the culture. While traditional methods have used a single wavelength to measure the maximum absorption of a particular hemoglobin species independently, spectral deconvolution and least squares fitting algorithms can be used to determine hemoglobin species concentrations relative to the total hemoglobin amount to better approximate the total composition of hemoglobin species in a sample [79]. While these methods are typically only utilized in hematology contexts, in *Plasmodium* culture studies, such measurements could be performed to monitor for variability in the O_2_-carrying state of hemoglobin in the RBCs in a culture experiment over the course of a longitudinal study. Such methods can be very insightful for helping to characterize the host hemoglobin protein itself, and its oxygenation state.

## Current state of assessment of organ-level *Plasmodium falciparum* biology

The study of *P*. *falciparum* biology from the human body to the laboratory must involve bridging the gap between both systems to better understand the biology of the parasite during a malaria infection, especially regarding within-host variability in O_2_. Neither the complexity of tissue structure nor the variability of physiological differences across niches of the human body are mimicked very well in our standard culture systems. Some advances along this front could include the development of organoid culture systems derived from direct organ sample harvests [10,12,13]. Although these studies aid in the understanding of parasite sequestration, there is a need to assess organ-level parasite biology in a more tractable system where PMR can be assessed alongside parasite genetics, epigenetics, gene expression, posttranslational modifications, and/or metabolic activity over time. Advances have been made in organoid culture systems for studying microbial pathogenesis, including for the study of apicomplexan parasites. Three-dimensional culturing with hepatic cell lines and other cell-types have been successfully utilized in the study of *Plasmodium* liver stage development [80,81]. In these studies, which did explore the effect of varying O_2_ concentrations, more parasite growth occurred under lower O_2_ concentrations, and this result was shown to be dependent on the up-regulation of HIF-1α genes in the host cell [82].

Another ex vivo organoid culture system being used to study microbial pathogenesis is for the study of diseases caused by vertical transmission through the placenta. These are placental organoid cultures derived from stem cells found in placental tissue, which have been shown to be beneficial in understanding these infections and could inform the understanding of placental malaria [83]. Making sure these organoids can be studied in the context of *P*. *falciparum* O_2_ availability would entail they be cultured under the physiological O_2_ tension characteristic of that organ system from which they are meant to model, which is not commonly done for existing cell lines derived from organs [84].

## Conclusions

**Fig 4:**
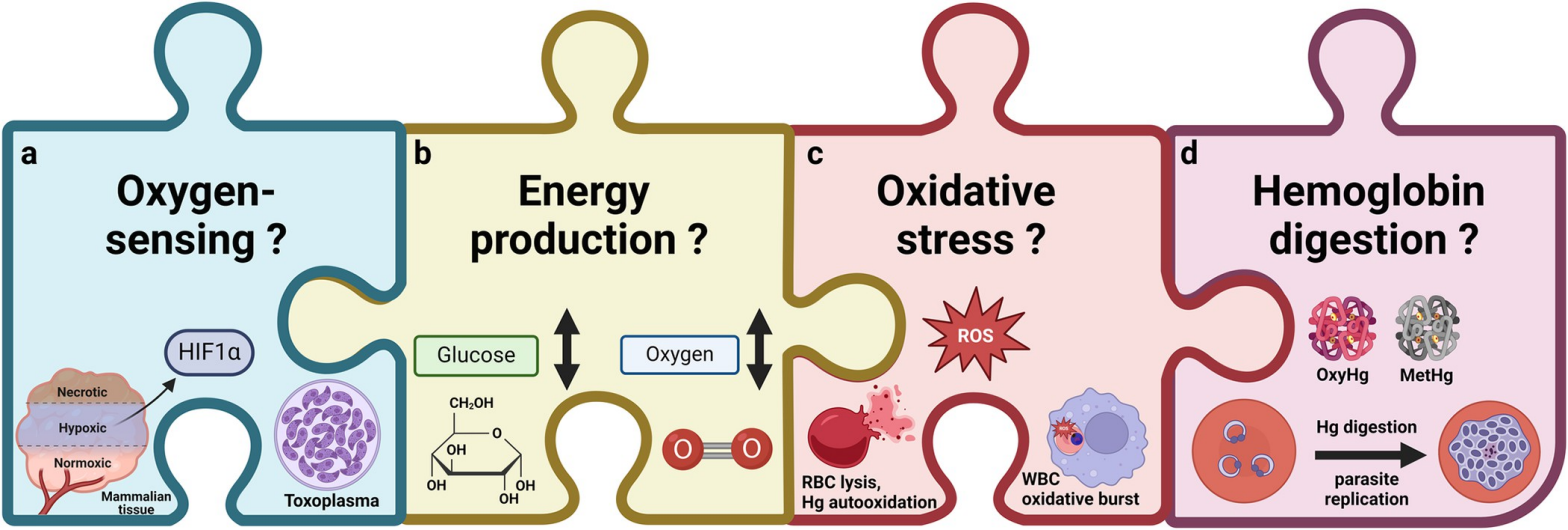
Pieces of the puzzle: possible mechanistic connections between O_2_ tension and *P*. *falciparum* asexual blood stage growth rate variation. **(a)** Mammalian cells and other apicomplexans such as *Toxoplasma* perform O_2_ sensing through HIFs and PHDs. While *Plasmodium* has a putative PHD B group gene, it is not known whether the O_2_-sensing function is active in *Plasmodium*. **(b)** *P*. *falciparum* exhibits plasticity in energy production across its hosts, relying primarily on glucose and glycolysis in the human host but requiring O_2_ and mitochondrial oxidative phosphorylation in the mosquito. Whether plasticity exists within the human host but between tissue sites with varying levels of O_2_ and/or glucose is unknown. **(c)** There are numerous sources of oxidative stress during a malaria infection including release of free heme during RBC lysis, auto-oxidation of hemoglobin, immune cell induced ROS production during oxidative burst, among others. ROS production is influenced by O_2_ availability and oxidative stress can impact *Plasmodium* intraerythrocytic development. **(d)** Finally, given that hemoglobin is a major source of nutrients for *Plasmodium*, the oxygenation state of hemoglobin could impact *Plasmodium*’s digestion and growth. Methemoglobin, a form of hemoglobin that cannot bind O_2_ and forms in conditions of oxidative stress, has been shown to bind to falcipain-2 at a higher affinity than oxidized hemoglobin, potentially leading to impacts in digestion and growth rate. HIF, hypoxia induction factor; PHD, prolyl hydroxylase; RBC, red blood cell; ROS, reactive oxygen species.

Elucidating the role of O_2_ tension on blood-stage *P*. *falciparum* growth will enhance our understanding of the parasite’s biology. There are many pieces to this puzzle, as depicted in **Fig 4**, that may or may not relate to the capacity of *P*. *falciparum* to survive at varying O_2_ tensions within the human body, including potential O_2_-sensing mechanisms, the parasite’s plasticity in energy production, the role of oxidative stress, and the parasite’s response to digesting different types of hemoglobin, all of which are worthy of further investigation. Open questions include the following:

- Does *Plasmodium* have an O_2_-sensing mechanism akin to that of *T*. *gondii*?
- Is mitochondrial OXPHOS used by the parasite in vivo during blood-stage development as a mechanism to help it compensate for energy deficits?
- Is the plasticity exhibited by *P*. *falciparum* an effect of genetic adaptations, epigenetic changes, gene expression changes, posttranslational modifications, or some combination of the above?
- To what extent does the oxygenation state of hemoglobin impact *P*. *falciparum*’s ability to digest it and subsequently grow and replicate?
- How much effect does O_2_ tension have on antimalarial susceptibility for *P*. *falciparum*?
- What is the dissolved O_2_ concentration that reaches RBCs in cultures gassed with 1% to 5% O_2_ and how well is this maintained over time?
- How much of an effect do different culture containment devices and gas administration methods have on dissolved O_2_ concentration?

Future work focused on these questions and others will improve our understanding of the complex relationship(s) between *P*. *falciparum*, its host cell, and O_2_.
